# Single-cell RNA sequencing reveals intratumoral heterogeneity and potential mechanisms of malignant progression in prostate cancer with perineural invasion

**DOI:** 10.3389/fgene.2022.1073232

**Published:** 2023-01-09

**Authors:** Bao Zhang, Shenghan Wang, Zhichao Fu, Qiang Gao, Lin Yang, Zhentao Lei, Yuqiang Shi, Kai Le, Jie Xiong, Siyao Liu, Jiali Zhang, Junyan Su, Jing Chen, Mengyuan Liu, Beifang Niu

**Affiliations:** ^1^ Department of Urology, Aerospace Center Hospital, Beijing, China; ^2^ ChosenMed Technology (Beijing) Co., Ltd., Beijing, China; ^3^ Computer Network Information Center, Chinese Academy of Sciences, Beijing, China; ^4^ University of the Chinese Academy of Sciences, Beijing, China

**Keywords:** prostate cancer with perineural invasion, intratumor heterogeneity, single-cell RNA sequencing, epithelial basal/intermediate cells, neural cells, progression, intercellular communication

## Abstract

**Background:** Prostate cancer (PCa) is the second most common cancer among men worldwide. Perineural invasion (PNI) was a prominent characteristic of PCa, which was recognized as a key factor in promoting PCa progression. As a complex and heterogeneous disease, its true condition is difficult to explain thoroughly with conventional bulk RNA sequencing. Thus, an improved understanding of PNI-PCa progression at the single-cell level is needed.

**Methods:** In this study, we performed scRNAseq on tumor tissues of three PNI-PCa patients. Principal component analysis (PCA) and Uniform manifold approximation and projection (UMAP) were used to reduce dimensionality and visualize the cellular composition of tumor tissues. The differently expressed genes among each cluster were identified by EdgeR. GO enrichment analysis was used to understand the roles of genes within the clusters. Pseudotime cell trajectory was used to reveal the molecular pathways underlying cell fate decisions and identify genes whose expression changed as the cells underwent transition. We applied CellPhoneDB to identify cell-cell interactions among the epithelial and neural cells in PNI-PCa.

**Results:** Analysis of the ∼17,000 single-cell transcriptomes in three PNI prostate cancer tissues, we identified 12 major cell clusters, including neural cells and two epithelial subtypes with different expression profiles. We found that basal/intermediate epithelial cell subtypes highly expressed PCa progression-related genes, including *PIGR*, *MMP7,* and *AGR2*. Pseudotime trajectory analysis showed that luminal epithelial cells could be the initiating cells and transition to based/intermediate cells. Gene ontology (GO) enrichment analysis showed that pathways related to cancer progressions, such as lipid catabolic and fatty acid metabolic processes, were significantly enriched in basal/intermediate cells. Our analysis also suggested that basal/intermediate cells communicate closely with neural cells played a potential role in PNI-PCa progression.

**Conclusion:** These results provide our understanding of PNI-PCa cellular heterogeneity and characterize the potential role of basal/intermediate cells in the PNI-PCa progression.

## Introduction

PCa is currently the most commonly diagnosed malignancy in the worldwide male population and one of the frequent leading causes of cancer-related death in men (Siegel et al., 2020). PNI is one of the characteristics of PCa and a prognostic factor in radical prostatectomy specimens. It is defined as cancer cell invasion in, around, and through nerves and is a well-established mechanism of tumor spread. In recent years, it has been demonstrated that PNI was associated with poor survival and increased risk for metastasis in PCa (Wu et al., 2019; Lin et al., 2020).

The majority of prostate cancers are carcinomas, which originate from epithelial cells. There is evidence implicating epithelial in prostate cancer (PCa) progression. Under certain physiological and pathological conditions, epithelial cells differentiate into mesenchyme, accompanied by changes in cell morphology and related genes (Brabletz et al., 2018), which is called epithelial mesenchymal transition (EMT). Emerging evidence demonstrates that the EMT process can promote tumor cell invasion and metastasis by loss of cell-cell connections and cell polarity (Dudas et al., 2020). Furthermore, many studies have reported the effects of PNI on patients’ recurrence, metastasis, and prostate cancer-specific survival. *In vitro* experiments have illustrated that prostate cancer cells in close contact with nerves exhibit a reduced rate of apoptosis (Deborde and Wong, 2017). A meta-analysis by Zhang et al. (2018) demonstrated that the presence of PNI in PCa was associated with a higher risk of biochemical recurrence, whether undergoing radical prostate prostatectomy or radiotherapy. Importantly, recent studies have identified several mechanisms by which neural infiltration promotes disease development and metastasis. These included PNI, which results in PCa-related axonotmesis, neurotmesis, and cancer cell proliferation and migration (Fromont et al., 2012; Zhang et al., 2016). Moreover, some studies have reported that the progression of cancer results from diverse signaling transduction between cancer cells and the neural microenvironment driven by chemokines, neurotrophic factors, and axon guidance molecules through ligand-receptor interactive mode. Specifically, monoamine oxidase A (MAOA), nerve growth factor (NGF), semaphorin4D (SEMA-4D), and C-X-C motif chemokine ligand 12 (CXCL12) were recently demonstrated to have an active role in promoting PNI in PCa and may be critical mediators in the pathogenesis of PNI (Zhang et al., 2008; Binmadi et al., 2012; Liss et al., 2014; Yin et al., 2021).

Although several molecular mechanisms have been discovered involving prostate tumor development and progression, most of these studies have focused on bulk tissue samples or cell populations. As PCa is a highly heterogeneous tumor, its true condition is difficult to explain thoroughly with conventional bulk RNA sequencing, which leaves a gap in our understanding of the mechanism underlying cancer progression at the single-cell level and hampers the development of novel therapeutic strategies for PCa treatment. scRNA-seq has become the primary tool for characterizing the transcriptomes in thousands of individual cells and provides unprecedented opportunities to understand the cellular heterogeneity and interaction of individual cells (Aibar et al., 2017; Kumar et al., 2018). Although this technology has been applied to several types of cancer in multiple cancers (Chen et al., 2021; He et al., 2021), there are few studies on PNI-PCa. Thus, further analysis of PNI-PCa at the single-cell level is essential for discovering the causes contributing to disease progression.

In this study, we conduct a comprehensive evaluation of prostate tumor tissues by scRNA-seq to analyze their cellular compositions and explore the specific cell subtypes associated with tumor progression of PNI-PCa. Our results indicated that the basal/intermediate epithelial cells are associated with the progression of PNI-PCa. Further, we showed that the interactions between basal/intermediate epithelial cell subtypes and neural cells subtypes through CXCL and CCL signaling network pathways promote tumor progression. These findings have important implications for our understanding of the mechanistic related to PNI-PCa progression and provide some new references for the therapeutic strategy for PNI-PCa.

## Materials and methods

### Samples collection

The tissue samples were obtained from three PNI-PCa patients who underwent surgical resection at the Department of Urology, Aerospace Center Hospital. All patients had lesions visible on preoperative Magnetic Resonance Imaging (MRI) or ultrasound and were later pathologically diagnosed as PCa with perineural invasion (Supplementary Figure S1). All patients provided informed consent and the study protocol permitted by the Ethics Committee of Aerospace Center Hospital.

### Single-cell preparation

The tumor tissues were dissociated using Multi-tissue dissociation kit 2 (Miltenyi) after being washed in ice-cold RPMI1640. DNase treatment was performed according to the manufacturer’s protocols. After removing erythrocytes (Miltenyi 130-094-183), cell count and viability were determined by Cell Analyzer (Countstar^®^ Rigel S2) with AO/PI reagent. Then debris and dead cell removal were decided on whether to be performed or not (Miltenyi 130-109-398/130-090-101). Finally, new cells were washed twice in the RPMI1640 and resuspended at 1 × 106 cells per ml in 1 × PBS and .04% bovine serum albumin.

### Single-cell RNA sequencing and data processing

Single-cell RNA-Seq libraries were prepared using SeekOne^®^ MM Single Cell 3′ library preparation kit Briefly, the appropriate number of cells were loaded into the flow channel of the SeekOne^®^ MM chip, which had 170,000 microwells and was allowed to settle in microwells by gravity. After removing the unsettled cells, sufficient Cell Barcoded Magnetic Beads (CBBs) were pipetted into the flow channel and allowed to settle in microwells with the help of a magnetic field. Next excess CBBs were rinsed out, and cells in the MM chip were lysed to release RNA, which was captured by the CBB in the same microwell. Then all CBBs were collected, and reverse transcription was performed at 37°C for 30 min to label cDNA with cell barcode on the beads. Further, Exonuclease I treatment was performed to remove unused primer on CBBs. Subsequently, barcoded cDNA on the CBBs was hybridized with a random primer which had reads 2 SeqPrimer sequences on the 5′ end and could extend to form the second strand DNA with cell barcode on the 3’ end. The resulting second strand DNA was denatured off the CBBs, purified, and amplified in polymerase chain reaction (PCR). The amplified cDNA product was then cleaned to remove unwanted fragments and added to a full-length sequencing adapter and sample index by indexed PCR. The indexed sequencing libraries were cleaned up with SPRI beads, quantified using quantitative PCR (KAPA Biosystems KK4824), and then sequenced (PE150) by Illumina NovaSeq 6,000 platform.

### Data processing of single-cell RNA-seq

The raw sequencing data were processed by fastp (version .20.0) (Chen et al., 2018) firstly to trim primer sequence and low-quality bases. And then, we used SeekOne®Tools to process sequence data and aligned it to human GRCh38 to obtain the gene expression matrix. We used Seurat (version 4.0.3) to filter low-quality cells. The UMI counts per cell should generally be above 300, and belonging to mitochondrial genes was also omitted. The remaining cells were used for the following analysis.

### Unsupervised clustering and markers identification

The Seurat package “NormalizeData” function was used to normalize the matrix data of single-cell counts, where gene expression for each cell was normalized by dividing the total expression counts, multiplied by a scale factor of 10,000, and then log-transformed. We conducted normalization and then performed principal component analysis (PCA) on the normalized expression matrix using highly variable genes identified by the Seurat R package (version 4.0.0) (Satija et al., 2015). Following the results of PCA, the top 30 PCs were selected with a resolution parameter equal to .8. The cell clusters were visualized using the uniform manifold approximation and projection (UMAP). The “FindAllMarkers” function in Seurat was used to identify differentially expressed marker genes for each cell type. Marker genes were selected as those with adjusted *p* values less than .05, average logFC larger than .25, and percentage of cells with expression higher than .25. Cell type identities were determined based on the expression of known markers in the Cell Marker database (Zhang et al., 2019).

### Differentially expressed genes and enrichment analysis

The differentially expressed genes DEGs were identified using the edgeR package and filtered by |log2FoldChange| ≥ 1 and FDR <.05. GO enrichment analysis of DEGs involved in the biological process was implemented by the “ClusterProfiler” package in R software.

### Cell-cell interactions

We used CellPhoneDB to infer cell-cell interactions, processed and normalized logarithmic counts, and cell type annotations were used as an input. To shrink down the most relevant interactions, specific interactions sorted by ligand/receptor expression were investigated in over 10% of the cells in a cluster, where the log2 mean expression of the ligand/receptor pair is greater than 0.

### Pseudotime trajectory analysis

Monocle (version 2.3.6) (Trapnell et al., 2014) was used to estimate lineage differentiation between cell populations using default parameters. First, a CellDataSet object was created with the parameter expressionFamily as negbinomial, then genes used for pseudotime ordering were selected using the ‘dispersionTable’ Function. The ‘DDRTree’ method was utilized for dimension reduction and cell ordering along the pseudo time trajectory. Branch analysis was performed with the branched expression analysis modeling (BEAM) function. The significantly changed genes in the branch point were clustered using “plot_genes_branched_pseudotime” functions according to the distinct patterns of gene expression change.

## Results

### scRNA-seq profiling and cell typing in PNI-PCa

**FIGURE 1:**
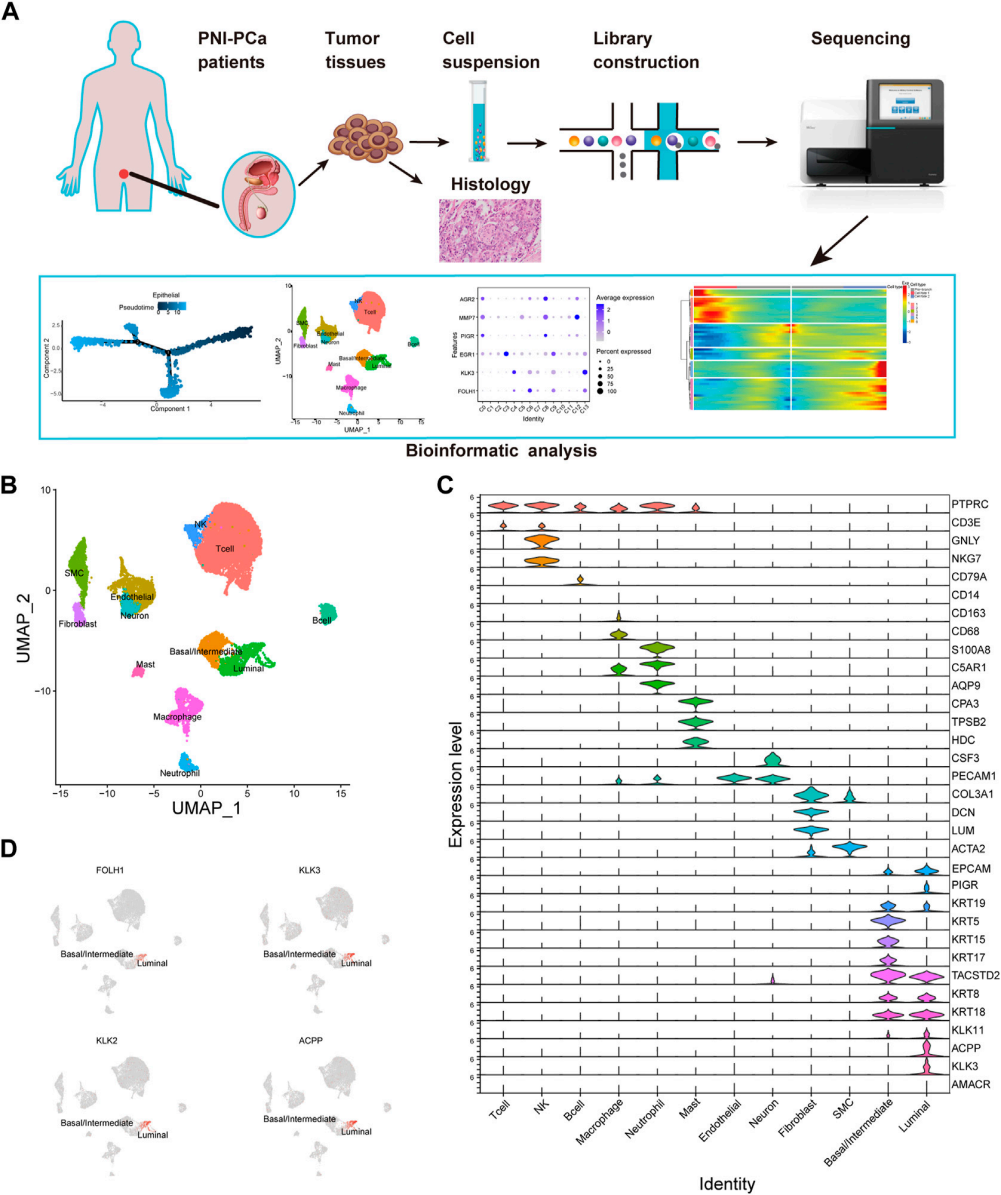
Diverse cell types in PNI-PCa were identified by single-cell sequencing. **(A)** Single-cell sequencing flow chart. **(B)** UMAP exhibits the cell clusters in PNI-PCa and different colors labeled for different cell types according to their featured gene expression profiles. **(C)** Violin plots show the expression levels of marker genes for each cell type. **(D)** UMAP shows the expression of prostate cancer-specific genes localized specifically to the luminal epithelial cells.

To understand PNI prostate tumor heterogeneity at single-cell resolution, we obtained tissue samples from patients diagnosed as PCa with perineural invasion and performed single-cell RNA sequencing. The study workflow is shown in Figure 1A. After data processing and normalization, we obtained 17,561 qualified cells for further analysis. Next, we examined the cellular composition with unbiased clustering across all cells by PCA and visualized by UMAP for Dimension Reduction (Figure 1B). To identify the different major cell types, we analyzed the expression level of the cluster-specific gene markers in each cluster identified by examining DEGs, allowing us to categorize these clusters into 12 main clusters: T lymphocyte, natural killer cell, B lymphocyte, macrophage, neutrophil, mast cell, endothelial cell, fibroblast, smooth muscle cell (SMC), neural cell and cell (Figure 1C). Strikingly, we found that the epithelial cells population was divided into two major subsets: luminal and basal/intermediate cells. Differential gene expression analysis results in epithelial cell subtypes showed that basal/intermediate cells exhibited much higher expression levels of basal markers, including *KRT5*, *KRT15*, and *KRT17*, compared with other clusters. The gene of *KLK11* was specifically expressed in the luminal cells, which could potentially be used as specific markers for the detection of luminal cells in PNI-PCa. An analysis of prostate cancer-specific gene expression levels in each cell type was performed to evaluate the malignancy of these cell clusters. We found that the prostate cancer-specific genes, including *KLK3*, *KLK2*, *FOLH1*, and *ACPP,* are expressed mainly in luminal cells (Figure 1D). Furthermore, we identified a subset of neural cells according to the high expression level of marker gene *CSF3* (Figure 1C). We further grouped neuronal cells into four clusters based on the scRNA-seq data (Supplementary Figure S2).

### Epithelial cell subtypes play a potential role in PNI-PCa progression

**FIGURE 2:**
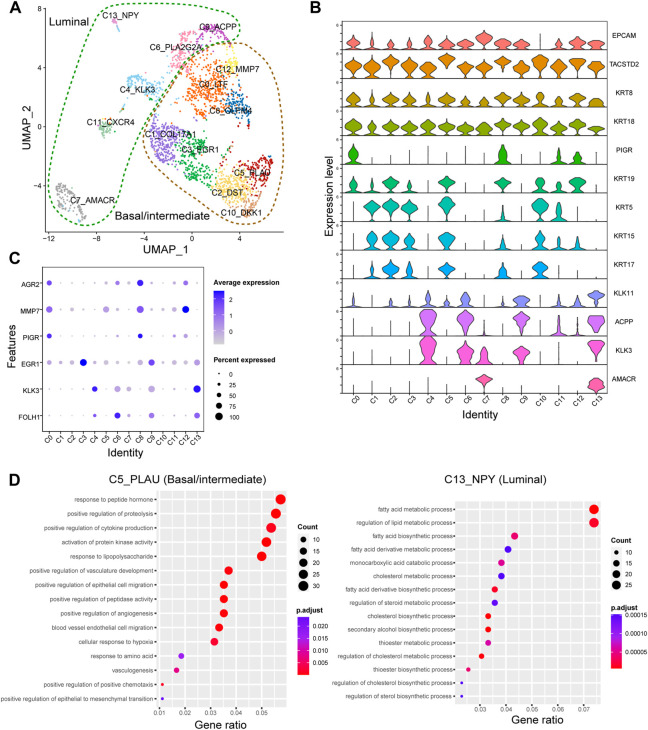
The epithelial cell type classifications in PNI-PCa tumor tissue. **(A)** Epithelial cells are divided into 13 different subgroups, UMAP of epithelial cells annotated by cell types. **(B)** Violin plots of marker genes across the epithelial cell subtypes. **(C)** The relative expression of *FOLH1*, *KLK3*, *EGR1*, *PIGR*, *AGR2,* and *MMP7* genes in different epithelial subgroups. **(D)** GO enrichment analysis for DEGs in basal/intermediate cell subtype C5 and luminal cell subtype C13, respectively.

To further explore the potential roles of epithelial cells in PNI-PCa, we performed a graph-based clustering analysis, and 14 clusters were identified (C0-C13, Figure 2A). To characterize these epithelial clusters, we used VlnPlot to examine the expression patterns of the specific marker genes individually. The results showed that the genes including *KRT5*, *KRT15*, *KRT19*, and *KRT17*, exhibited higher expression levels in C0, C1, C2, C3, C5, C8, C10, and C12 (Figure 2B), which were identified as basal/intermediate cells subtypes. Other epithelial cells, including C4, C6, C7, C9, C11, and C13, were identified as luminal cells based on the expression of specific markers *KLK3* and *ACPP*.

To identify the transcriptional states of epithelial cell subtypes, we used the edgeR package to examine gene expression patterns in these cells. The luminal cells, including C4, C6, C7, C9, and C13, showed significantly higher levels of expression of PCa markers genes *FOLH1* and *KLK3* (Figure 2C). Compared with other epithelial clusters, the luminal cell subtypes of C9 exhibited a higher expression level of *EGR1.* It has been reported that the expression level of *EGR1* was increased in PCa and related to the occurrence, progression, and metastasis of PCa (Li et al., 2019). The human anterior gradient-2 (AGR2), a urinary marker for PCa, was highly expressed in C0, C8, and C12. It is worth noting that *PIGR* and *MMP7* significantly high expressions were detected in the basal/intermittent C0 and C8. In previous studies, *PIGR* has demonstrated a role in promoting cellular transformation and proliferation. It has also been reported that *MMP7* can promote PCa by inducing epithelial-mesenchymal transition (Yue et al., 2017; Zhang et al., 2017). These results suggested that basal/intermediate cell subtypes may be involved in tumor progression. Next, GO enrichment analysis was performed to examine further the potential roles of each epithelial cluster in PNI-PCa progression. We found that the genes upregulated in the luminal cell subtype of C13 were mainly related to metabolic processes involved in cancer progressions, including fatty acid metabolism, lipid metabolism, and steroid biosynthesis (Figure 2D), indicating that luminal cells were indeed malignant. Additionally, the genes upregulated in basal/intermediate cell subtypes C5, were associated with tumor migration processes such as epithelial-mesenchymal transition, vasculogenesis, and endothelium development (Figure 2D), indicating that basal/intermediate cell subtypes were associated with PNI-PCa progression.

**FIGURE 3:**
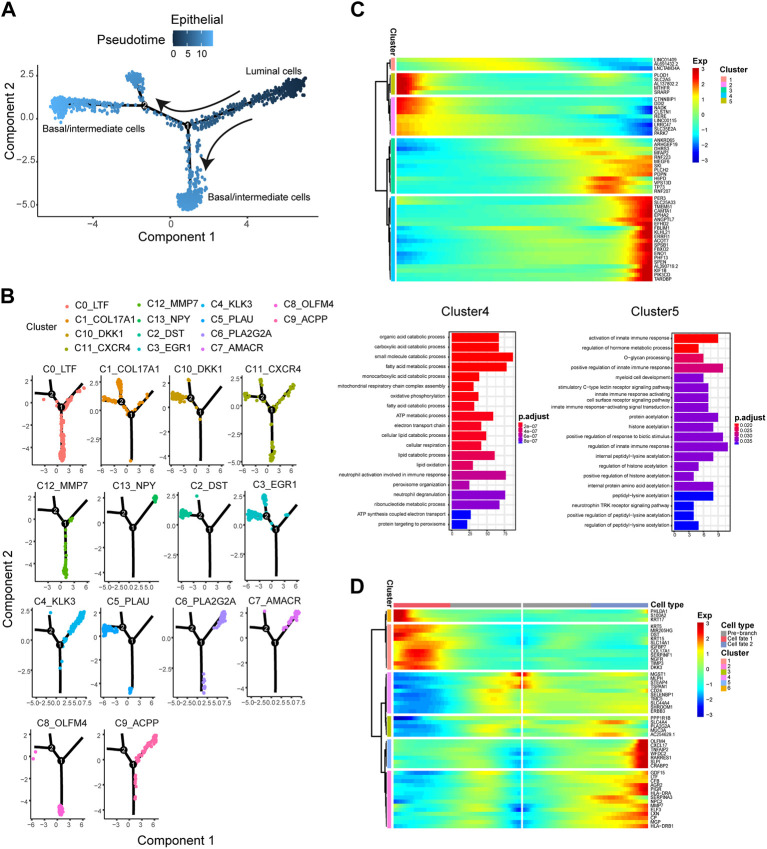
The pseudotime trajectory analysis of epithelial cells and identifying genes varied during the trajectory. **(A)** Differentiation trajectory of epithelial cells constructed by Monocle. **(B)** The epithelial cell subtypes differentiation trajectory plot. **(C)** Upper panel: The top 50 DEGs with the most significant expression level change over the pseudotime trajectory were classified into 5 clusters. Lower panel: GO molecular function enrichment heatmap of Cluster 4 and 5. **(D)** The heatmap shows the expression patterns of the top 50 significant genes in branched expression analysis modeling.

To explore the relationship among these epithelial cell subtypes and identify the association between the genes associated with epithelial cell reprogramming, we pooled the epithelial cell subtypes and constructed a transcriptional trajectory of these cells on a pseudoprime scale. Analysis of the pseudotemporal trajectory presented two prominent paths for the epithelial cell subtypes (Figure 3A). The luminal cell subtypes, including C4, C6, C7, C9, and C13, were shown at the beginning of the trajectory (Figure 3B). The base/intermediate cells subtypes of C0, C8, and C12 appeared at the end of the trajectory branch 1, and branch 2 is composed of other basal/intermediate cells, including C1, C2, C3, C5, and C10 (Figure 3B). These results indicated that luminal epithelial cells could be the initiating cells and transition to based/intermediate cells.

We then analyzed the changes in the dynamic expression of genes along the trajectory (Figure 3C). We identified some of the genes with the most significant changes in expression, including *EPHA2*, *PDPN*, *SPSB1*, and *FBXO2*, which have been reported to be related to multiple cancer metastasis or cell movement (Feng et al., 2014; Sun et al., 2018; Hu et al., 2020). In addition, from the initial stage of the pseudotime trajectory (luminal cell subtypes) to the terminal stage (basal/intermediate cell subtypes), we found some genes with pseudotemporal expression patterns clustered into five specific clusters. Go enrichment analysis was performed to reveal the different functions of these gene clusters. We identified genes cluster 4, which were highly expressed at the end stage of the pseudotime trajectory (Figure 3C) and mainly involved in PCa progression-related processes, including fatty acid metabolic process, organic acid catabolic process, and lipid catabolic process. Otherwise, the genes cluster 5 were highly expressed at the beginning stage of the pseudotime trajectory and enriched in activation of the innate immune response (Figure 3C).

Furthermore, BEAM analysis was performed to identify the cell cluster marker genes that change as epithelial cell subtypes pass from the luminal to the basal/intermediate stage. We performed a branched heatmap to show the expression dynamic of the top 50 significant genes in different cell fate branches (Figure 3D). From pre-branch (luminal cell subtypes) to cell fate 2 (Basal/intermediate cell subtypes including C1, C2, C3, C5, and C10), we found that two gene clusters (cluster 2 and cluster 5) were highly expressed in fate2. In the two gene clusters, some genes associated with tumorigenesis and cancer progression, such as *AGR2*, *MMP7*, *LXN*, *PIGR*, and *CRABP2,* were identified (Figures 3D). Additionally, from pre-branch (luminal cell subtypes) to cell fate 1 (basal/intermediate cell subtypes including C0, C8, and C12), gene cluster 1 and cluster 6 were highly expressed in fate1. We demonstrated that the expression level of *KRT17*, *S100A2*, and *KRT15* was significantly higher in cluster 1 (Figure 3D). It had been reported that these genes were closely related to tumor progression and metastasis (Wang et al., 2019; Rao et al., 2020; Zhang et al., 2022).

### The communication between epithelial and neural cells is associated with the progression of PNI-PCa

**FIGURE 4:**
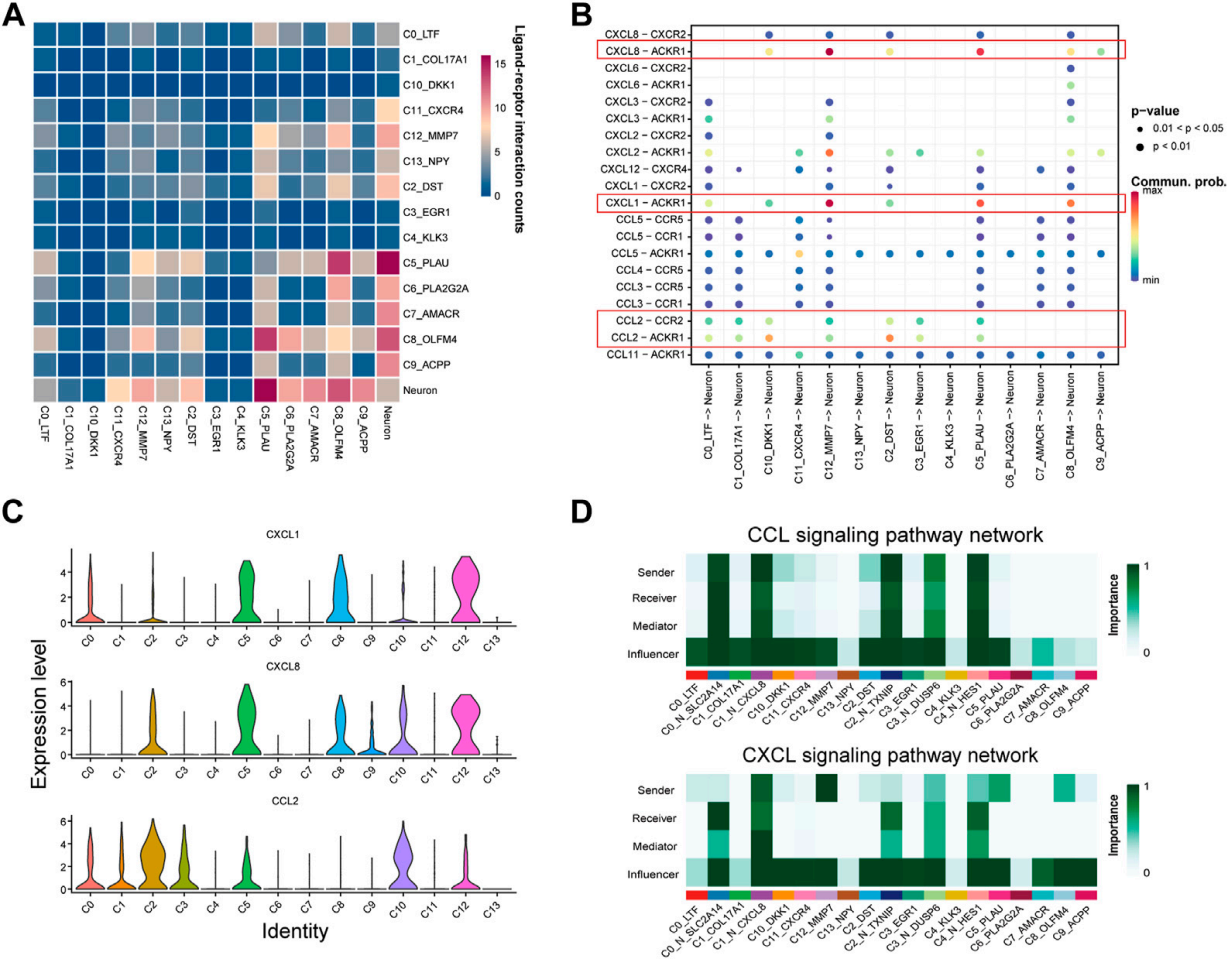
Cell interaction networks in PNI-PCa. **(A)** Heatmap shows the number of potential ligand-receptor pairs between epithelial cell subtypes and neural cell sells predicted by Cellphone DB. The more red the color, the stronger cell-cell communication. **(B)** Bubble plot of ligand-receptor interactions between epithelial cell subtypes and neural cells, where the bubble size represents the *p*-value (the *p*-value of all bubbles is less than .05), the bigger the bubble, the smaller the *p*-value. The color represents cell interaction strength, the redder the color, the stronger the interaction. **(C)** Bar chart showing the expression levels of CCL2, CXCL1, and CXCL8 in each epithelial cell subgroup. **(D)** The communication probability of neural and epithelial cells on the CXCL and CCL signaling pathways.

Studies have shown that PNI in the tumor can contribute to the prognosis of PNI-PCa. Emerging evidence also suggested that the interaction between cells played a crucial role in tumor development. We hypothesized that the interactions between epithelial cells, especially basal/intermediate cells and neural cells, might facilitate the malignant growth of PNI-PCa. Based on this assumption, we characterized cell-cell communication mediated by ligand-receptor interactions between neural and epithelial cells in tumors using scRNA-seq data. Our results showed that there were significant interactions between epithelial cell subtypes and neural cells, and these subtypes of the epithelial cell were predominantly basal/intermediate cells (Figure 4A). The attraction strengths of ligand-receptor pairs between the epithelial and neural cells were calculated to infer molecular interactions mediating cell-cell interactions. Among the ligand-receptor pairs pertaining to epithelial and neural cells, CXCL1-ACKR1 and CXCL8-ACKR1 were significantly enriched in basal/intermediate C5, C8, and C12 (Figure 4B). As members of the CXC chemokine family, CXCL1 and CXCL8 have reported an association with the development of multiple cancers (Chen et al., 2020; Unver, 2021). In addition, another ligand-receptor pair involving interactions between epithelial cell subtypes and neural cell subsets were CCL2-CCR2 and CCL2-ACKR1, which mainly enriched in basal/intermediate C2 and C10 (Figure 4B). Compared with other epithelial cells, higher expression of CCL2 was detected in basal/intermediate C2, C3, and C10 (Figure 4C). It has been reported that the chemokine ligand CCL2 and its receptor ACKR1 were implicated in the initiation and metastasis in multiple cancers, which suggested the potential role of the interaction between basal/intermediate epithelial and neural cells in PNI-PCa progression.

We further dissected CXCL and CCL signaling of communication probability between neural cell and epithelial cell subtypes. In the CXCL signaling network, basal/intermediate cell subtypes C5, C8, and C12 were the strong senders, all the neural cells were receivers, and the neural cell subtypes, including C0_N_SLC2A14 and C4_N_HES1, were most abundant (Figure 4D). For the CCL signaling network, basal/intermediate cell subtypes C2 and C10 were the major signal senders, and all the neural cell populations were the signal receivers (Figure 4D). Collectively, our results suggested that the cells communicate between basal/intermediate cell subtypes and neural cells through the CXCL and CCL signaling network, which may be one of the critical factors in promoting cancer progression and metastasis in PNI-PCa.

## Discussion

PNI is the process in which cancer cells penetrate along the neural commonly observed in PCa. In recent years, PNI has been confirmed to be associated with the progression and metastatic processes of PCa (Jia et al., 2017; Delahunt et al., 2020). Some studies also have revealed several molecular mechanisms of their influence on PCa progression at bulk tissue samples and cell populations level (Alajati et al., 2020; Pomerantz et al., 2020). However, the underlying molecular mechanisms affecting the progression of PNI-PCa at the single-cell level have been unclear. In our study, we performed scRNA-seq using tumor tissues obtained from PNI-PCa patients, and 12 cell types were identified, including two types of epithelial clusters and neural clusters. We found that basal/intermediate cell subtypes play an important role in PNI-PCa progression. In addition, the intercellular communication between basal/intermediate cells and neural cells *via* CCL and CXCL signalling pathways may be a possible molecular mechanism for PNI-PCa progression. Our results provide a more holistic interrogation of PNI-PCa progression at an unprecedented single-cell resolution. As far as we know, little has been reported about this aspect of the study. Several lines of evidence support that most prostate cancer originates from luminal cells (Chua et al., 2018; Guo et al., 2020). Consistent with previous studies, our results showed that prostate cancer-specific genes are mainly expressed in luminal cells. A previous study indicated that basal cells from human primary benign prostate tissue could cause PCa in immunodeficient mice (Goldstein et al., 2010), implying the potential roles of basal cells in tumorigenesis. In this study, we found that basal/intermediate cell populations were closely related to the progression of PNI-PCa. Compared to other luminal cell subtypes, basal/intermediate cell subtypes C8 has a higher expression level of *AGR2*, which was reported to be overexpressed in PCa compared to benign tissue and played an essential role in cancer spread (Takabatake et al., 2021; Maarouf et al., 2022). Furthermore, our results revealed that some genes associated with the migration and development of PCa (Chen et al., 2016; Yue et al., 2017), including *MMP7* and *PIGR,* were highly expressed in basal/intermediate cell subtypes of C0 and C12. GO enrichment analysis also showed that pathways related to cancer progression were significantly enriched in basal/intermediate cells. These results suggested the pivotal roles that basal/intermediate cells play in PNI-PCa progression.

To further investigate this, we next performed pseudotime analysis on epithelial cells. The results demonstrated that luminal cells transform into basal/intermediate cells as cancer progresses. Notably, we identified that *ENO1* and *EPHA2* were highly expressed at the end of preudotime trajectorypse. *EPHA2* is a member of the Eph family of receptor tyrosine kinases (RTK), expressed in most epithelial cells and associated with aggressive behavior in PCa(Kurose et al., 2019). In multiple malignancies, the expression of α-enolase (*ENO1*) is related to the progression and metastasis of the tumor (Hou et al., 2021; Li et al., 2021). Together, the results above demonstrated the effect of basal/intermediate epithelial cell subtypes on PNI-PCa progression.

PNI is a form of cancer progression where cancer cells invade along nerves. Prostate cancer cell interaction with nerves induces a neurogenesis response that promotes the initiation and metastasis of PCa (Magnon et al., 2013). The study of the PNI mechanism shows that ligand-receptor interactions between nerves and cancer cells can promote the process of PNI(Marchesi et al., 2010; Binmadi and Basile, 2011). In our results, we identified that basal/intermediate cell subtypes were the cells that communicate most closely with nerve cells through CCL2-ACKR1, CXCL1-ACKR1, and CXCL8-ACKR1 signaling. CCL2 is overexpressed in various cancers, including glioma, prostate, and breast (Tsaur et al., 2015; Yang et al., 2021; Zhong et al., 2021). Recent studies have implicated the role of chemokine ligand CCL2 and its receptor CCR2 in the progression of multiple cancers (Fang et al., 2021; Xu et al., 2021). Furthermore, some studies have found that CCL2, with its receptor CCR2, played a significant role in PNI in PCa and promoted the metastasis of PCa, using both animal models and a review of human surgical specimens (Ding et al., 2015; He et al., 2015). In our study, we also identified that CXCL1 and CXCL8 were significantly stronger expressed in basal/intermediate cells. It is well known that these chemokines are widely expressed on cancer cells and associated with malignant tumors. Several studies have demonstrated that CXCL1 and CXCL8, as well as their cognate receptors, can mediate tumor growth, proliferation, and metastasis of malignant cells, including PCa (Adekoya and Richardson, 2020; Łukaszewicz-Zając et al., 2020). This evidence suggested that communication between basal/intermediate cells and neural cells plays a critical role in promoting the development and progression of PNI-PCa.Notably, it has been reported that neural cells were related to resistance to antiandrogen in patients with PCa. Li et al. (2016) found that neuronal genes were highly expressed during prostate cancer progression, resulting in the development a neuronal phenotype of PCa. Moreover, their results also revealed that the amount of neuroendocrine cells increases during antiandrogen PCa, leading to therapy resistance. Although many existing studies have shown that the relationship between basal cells and PCa (Xin, 2019), the effects of basal cells on therapy resistance are still not fully understood. In a recent study, the basal cell gene-expression profile was found to be significantly enriched in castration-resistant and advanced prostate cancers (Zhang et al., 2016), which indicated basal cells might play a potential role in the development of resistance to anti-androgens in PCa. In a study published by Marhold et al. showed that basal cells were involved in primary resistance to androgen receptor inhibition in the TRAMP mouse model (Marhold et al., 2022). In addition, increasing evidence suggested that androgen signaling through the androgen receptor (*AR*) contributes to the promotion of castration-resistant prostate cancer (*CR*PC) (Zhou et al., 2017). Olar et al. (2014) found that increased perineural invasion diameter was associated with higher levels of AR in PC. These results seem to indicate a potential role of the PNI in the development of CRPC. However, more in-depth studies are required to elucidate these issues.

Although these studies reveal important discoveries, several limitations need to be considered. The small number of patients and samples is a limitation of this study. Another limitation of the current study is lacking of *in vitro* experiments to provide a deeper mechanistic investigation. Thus, further studies are required.

In conclusions, we systemically characterize the heterogeneity and gene expression landscapes of diverse cell types residing in PNI-PCa by using a single-cell RNA sequencing approach. Importantly, we revealed that the intercellular communication between basal/intermediate epithelial cell subtypes and neural cells through the ligand-receptor interaction pathway could affect the progression of PNI-PCa. These findings have important implications for our understanding of the mechanistic related to PNI-PCa progression and provided some new references for the therapeutic strategy for PNI-PCa.

## Data Availability

The datasets presented in this study can be found in online repositories. The names of the repository/repositories and accession number(s) can be found below: The raw sequencing data from this study were uploaded to the Genome Sequence Archive (GSA, https://bigd.big.ac.cn/gsa/); with accession number PRJCA013564.

## Supplementary material

SUPPLEMENTARY FIGURE S1
**(A–C)** Pathological results revealed perineural invasion of the prostate cancer cells (hematoxylin and eosin staining) in three PCa patients.

SUPPLEMENTARY FIGURE S2 Five subtypes generated from neural cells are demonstrated by UMAP.
