# Supplementary figures and images for: Single-cell RNA sequencing reveals intratumoral heterogeneity and potential mechanisms of malignant progression in prostate cancer with perineural invasion
Source: Front Genet. 2023 Jan 9;13:1073232. doi: 10.3389/fgene.2022.1073232 (PMC9875799; doi:10.3389/fgene.2022.1073232)

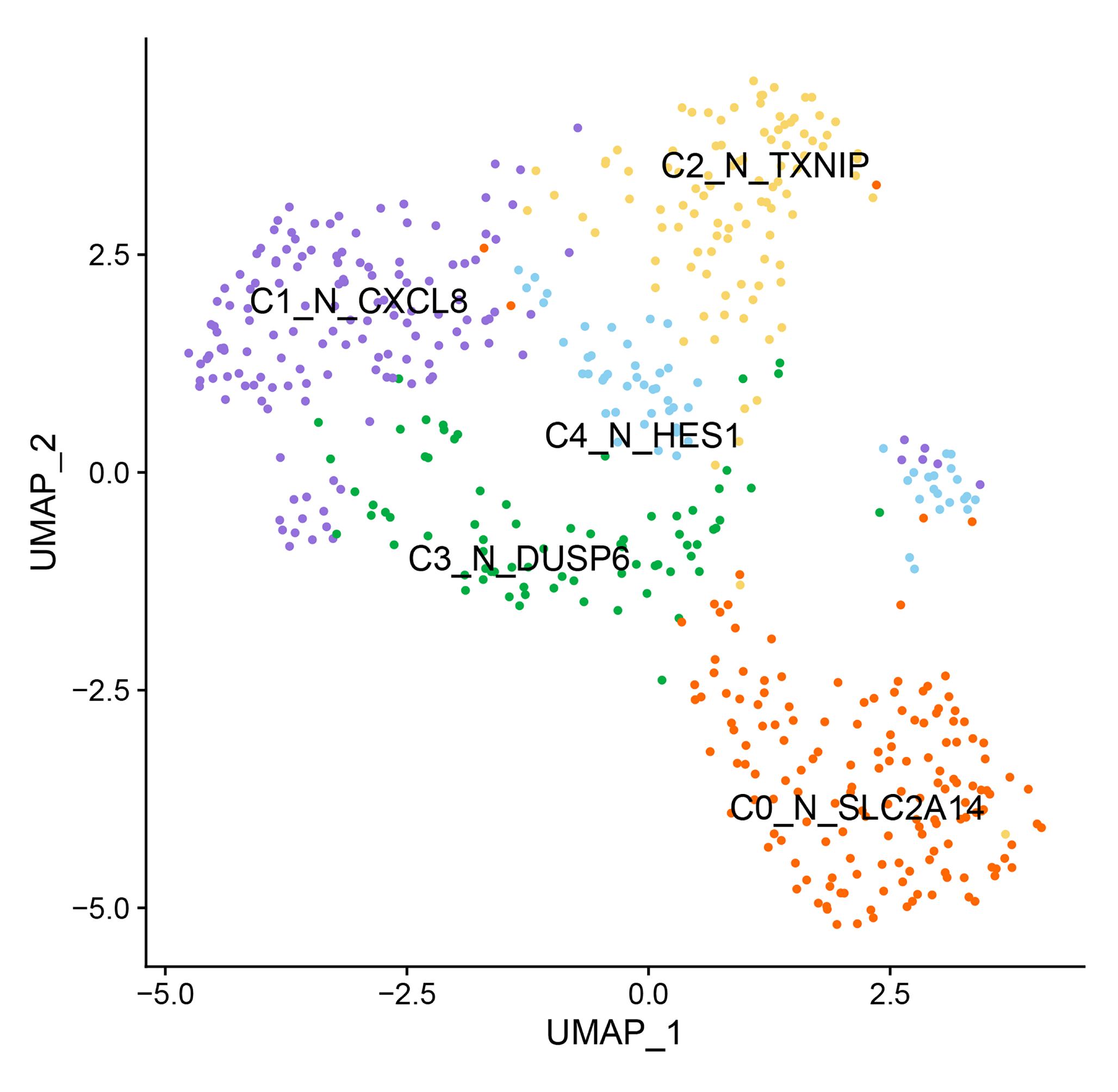

Supplement: Supplementary file 1 [file Image2.TIF]

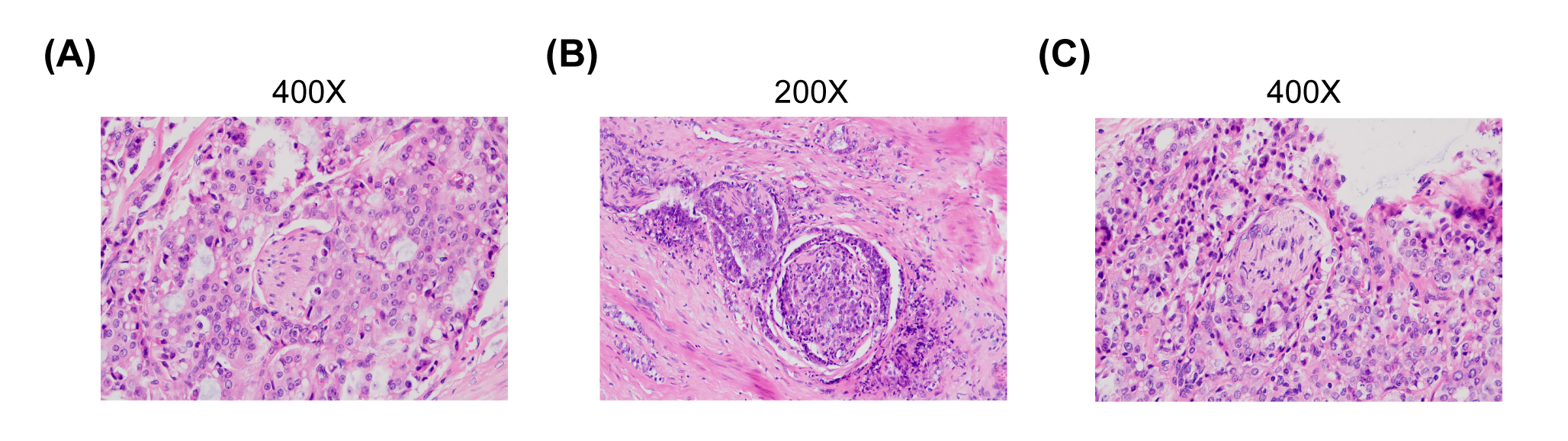

Supplement: Supplementary file 2 [file Image1.TIF]
